# 2476. Universal detection of healthcare-related bacterial transmissions using *de novo* assembly with short-read whole genome sequencing

**DOI:** 10.1093/ofid/ofad500.2094

**Published:** 2023-11-27

**Authors:** Theodore R Pak, Meghan Baker, Rebecca McSweeney, Kenneth E Sands, Julia Moody, Selsebil Sljivo, Amanda Isaacs, Edward J Septimus, Micaela H Coady, Russell Poland, Eunice Blanchard, Brandon Carver, Kimberly N Smith, Laura E McLean, Jeffrey Guy, Susan S Huang, Yonatan H Grad

**Affiliations:** Massachusetts General Hospital, Boston, Massachusetts; Brigham and Women's Hospital, Boston, MA; Harvard T.H. Chan School of Public Health, Boston, Massachusetts; HCA Healthcare, Nashville, Tennessee; HCA Healthcare, Nashville, Tennessee; Harvard Medical School and Harvard Pilgrim Health Care Institute, Boston, Massachusetts; Harvard Medical School and Harvard Pilgrim Health Care Institute, Boston, Massachusetts; Texas A&M University System Health Science Center, Houston, Texas; Harvard Medical School and Harvard Pilgrim Health Care Institute, Boston, Massachusetts; HCA Healthcare, Nashville, Tennessee; HCA Healthcare, Nashville, Tennessee; HCA Healthcare, Nashville, Tennessee; HCA Healthcare, Nashville, Tennessee; HCA Healthcare, Nashville, Tennessee; HCA Healthcare, Nashville, Tennessee; University of California, Irvine, Irvine, California; Harvard Chan School of Public Health, Boston, Massachusetts

## Abstract

**Background:**

Whole genome sequencing (WGS) is the gold standard for molecular evidence of pathogen transmission. To date, most WGS investigations within healthcare settings have used low cost short-read technologies (e.g., Illumina), aligning reads to a high quality reference genome. However, this relies on either public repositories having closely-related high quality references, which is not guaranteed for arbitrary clinical isolates, or additional long-read sequencing. *De novo* assembly avoids reliance on references, but the utility of fragmented assemblies of short reads is not well-established in hospital outbreak investigations.

**Methods:**

24 clusters of clinical isolates were identified by automated and routine methods at 17 U.S. hospitals and underwent paired-end WGS on Illumina platforms, followed by two parallel analyses. For a reference-free analysis, *de novo* assembly was performed with Shovill, assemblies were clustered with Mash, core-genome alignments were created with Harvest, and finally single nucleotide polymorphism (SNP) distances were visualized using the PathoSPOT phylogenomics toolkit to highlight likely transmission events. For a reference-guided analysis, references were selected for each isolate using StrainGST, and SNPs called by read alignment were used to define phylogenomic distances. Finally, conclusions from both analyses were compared.

**Results:**

92 bacterial isolates underwent WGS, of which 86 (93%) completed *de novo* assembly and passed quality control, comprising 10 isolates of *Acinetobacter baumanii*, 4 of *Klebsiella oxytoca*, 46 of *Klebsiella pneumoniae*, and 26 of *Staphylococcus aureus*. Reference-free analysis with PathoSPOT identified close relatedness consistent with transmission in 13 of 24 clusters (54%), while the reference-guided analysis identified the same in 12 clusters (50%). Overall, 21 clusters (88%) showed concordance between reference-free and reference-guided methods. Cohen’s kappa indicated substantial agreement (0.75; 95% CI, 0.49–1.0).

**Conclusion:**

*De novo* assembly of short reads is tenable for WGS investigation of hospital outbreaks, elides the need for reference genomes or long-read sequencing, and compares favorably with SNP calling via read alignment for detecting likely healthcare-related transmissions.

**Disclosures:**

**Laura E. McLean, M.Ed.**, HCA Healthcare: Stocks/Bonds **Susan S. Huang, MD MPH**, Medline Industries, Inc: Conducted studies whereby participating nursing homes and hospital patients received cleaning & antiseptic products|Xttrium Laboratories: Conducted studies where participating nursing homes and hospital patients received antiseptic products **Yonatan H. Grad, MD, PhD**, Day Zero Diagnostics: Board Member|GSK: Advisor/Consultant

